# Does canopy nitrogen uptake enhance carbon sequestration by trees?

**DOI:** 10.1111/gcb.13096

**Published:** 2015-12-14

**Authors:** Richard K. F. Nair, Micheal P. Perks, Andrew Weatherall, Elizabeth M. Baggs, Maurizio Mencuccini

**Affiliations:** ^1^ School of GeosciencesUniversity of EdinburghCrew BuildingEdinburgh, MidlothianEH9 3FFUK; ^2^ Forest ResearchNorthern Research StationRoslin, MidlothianEH25 9SYUK; ^3^ National School of ForestryUniversity of CumbriaAmblesideLA22 9BBUK; ^4^ Institute of Biological and Environmental SciencesUniversity of AberdeenZoology Building, Tillydrone AvenueAberdeenAB24 2TZUK; ^5^ Institució Catalana de Recerca i Estudis AvançatsCentre for Ecological Research and Forestry Applications, Cerdanyola del VallèsBarcelona08193Spain

**Keywords:** ^15^N labelling, C sequestration, canopy fertilization, canopy nitrogen uptake, isotope trace, Nitrogen deposition, *Picea sitchensis*, soil fertilization

## Abstract

Temperate forest ^15^N isotope trace experiments find nitrogen (N) addition‐driven carbon (C) uptake is modest as little additional N is acquired by trees; however, several correlations of ambient N deposition against forest productivity imply a greater effect of atmospheric nitrogen deposition than these studies. We asked whether N deposition experiments adequately represent all processes found in ambient conditions. In particular, experiments typically apply ^15^N to directly to forest floors, assuming uptake of nitrogen intercepted by canopies (CNU) is minimal. Additionally, conventional ^15^N additions typically trace mineral ^15^N additions rather than litter N recycling and may increase total N inputs above ambient levels. To test the importance of CNU and recycled N to tree nutrition, we conducted a mesocosm experiment, applying 54 g N/^15^N ha^−1^ yr^−1^ to Sitka spruce saplings. We compared tree and soil ^15^N recovery among treatments where enrichment was due to either (1) a ^15^N‐enriched litter layer, or mineral ^15^N additions to (2) the soil or (3) the canopy. We found that 60% of ^15^N applied to the canopy was recovered above ground (in needles, stem and branches) while only 21% of ^15^N applied to the soil was found in these pools. ^15^N recovery from litter was low and highly variable. ^15^N partitioning among biomass pools and age classes also differed among treatments, with twice as much ^15^N found in woody biomass when deposited on the canopy than soil. Stoichiometrically calculated N effect on C uptake from ^15^N applied to the soil, scaled to real‐world conditions, was 43 kg C kg N^−1^, similar to manipulation studies. The effect from the canopy treatment was 114 kg C kg N^−1^. Canopy treatments may be critical to accurately represent N deposition in the field and may address the discrepancy between manipulative and correlative studies.

## Introduction

Large differences exist among some estimates of the effect of nitrogen (N) deposition (N_DEP_) on temperate forest carbon (C) uptake in the northern hemisphere. These forests are a net C sink [0.6–0.7 Pg C yr^−1^ (Goodale *et al*., 2002)] and are also typically nitrogen limited (Vitousek *et al*., 2002; Lebauer & Treseder, 2008). Anthropogenic inputs of N from the atmosphere (Vitousek *et al*., 1997; Holland *et al*., 1999; Galloway *et al*., 2004) may enable extra C uptake (Thornley & Cannell, 1996) in these regions. However, estimates of C uptake due to N_DEP_ (henceforth referred to as ΔC/ΔN) from N additions tend to be low. Stable isotope tracer experiments where ^15^N is directly applied to the forest floor find that soil, litter and microbial biomass (SMB) are major sinks of N (70–80% of N_DEP_), while only ~20% of this ^15^N can be traced into trees and even less (< 5%) into woody components (Nadelhoffer *et al*., 1999; Templer *et al*., 2012a). As little N is recovered from high C/N biomass, the implied ΔC/ΔN is around 50 kg C kg N^−1^, which equates to only around 20% of net C uptake (Nadelhoffer *et al*., 1999). Other methodologies scaling measurements such as canopy N budgets or photosynthetic rates also tend to give low estimates of ΔC/ΔN [e.g. De Vries *et al*., 2006; Fleischer *et al*., 2013)], and one particular ^15^N amendment and modelling synthesis from Harvard Forest (Currie *et al*., 2004) found a ΔC/ΔN effect as small as < 5 kg C kg N^−1^, with most N for forest growth derived from natural abundance mineral soil as opposed to ^15^N deposition inputs applied to the soil.

Several correlational studies have since produced higher ΔC/ΔN estimates based on relationships between N_DEP_ and various indices of forest productivity. This was first raised by Magnani *et al*. (2007) who, from an estimate of C accumulation based on a comparison of European forest net ecosystem productivity found a strong influence of total (i.e. wet and dry) N_DEP_ at the continental scale, indicating an effect around 120–150 kg C kg N^−1^ (Magnani *et al*., 2008) when revised. These findings have variously been attributed to covariance of C uptake and N deposition with factors such as soil N capital and site history (De Vries *et al*., 2008; Högberg, 2012), climate or dry N_DEP_ contributions (De Vries *et al*., 2008; Sutton *et al*., 2008). Other studies using similar methodologies at the continent (Thomas *et al*., 2009) or country scale (Etzold *et al*., 2014; Ferretti *et al*., 2014) using forest inventory data have since implied similarly large effects. For such a large sink, N_DEP_ needs to be accumulated in trees and sequestered in high C/N, long‐lived bolewood (Townsend *et al*., 1996), which contradicts the results of manipulative experiments and ^15^N budgets.

An alternative explanation for these differences is that important real‐world processes included in correlative studies are not accurately represented in conventional deposition experiments. ^15^N amendment experiments (e.g. Nadelhoffer *et al*., 1999) typically apply ^15^N directly to the soil, and, while uptake of N by foliage has been considered for some almost 30 years (see early work; Garten *et al*., 1988; Hanson & Garten, 1992; Norby *et al*., 1989), such studies assume interactions with the canopy are of minimal importance. However, canopy N uptake (CNU) is now well documented (Sparks, 2009) and if substantial amounts of N are obtainable across leaf or branch surfaces this may substitute for, or supplement nutrition by soil pathways. Soil‐targeted studies could therefore underestimate the total ΔC/ΔN effect due to their assumption that only minimal amounts of N can be acquired by the canopy. CNU may also increase allocation to wood to 10 or 15% of total N_DEP_ (Sievering, 1999), which may influence the strong woody response implied by growth rates of some species across North America (Thomas *et al*., 2009). When incorporated into a recent modelling study, Dezi *et al*. (2010) found that forest management, CNU and induced changes in litter quality significantly raised the predicted ΔC/ΔN effect up to 121 kg C kg N^−1^.

Various potential mechanisms for CNU have been suggested, across both foliage and twigs, via ion exchange (Bowden *et al*., 1989; Boyce *et al*., 1996; Sparks, 2009) and simple diffusion (Klemm *et al*., 1989). These may vary in their absorption of specific N species (Wilson & Tiley, 1998) due to varying transport and metabolic costs to reduce N for biological incorporation, as well as internal cell N concentrations, and varying in their degree of incorporation into actively cycling pools within the plant (Sparks, 2009). In forests, estimates of CNU can be high, and some canopy N budgets (N_DEP_ – throughfall) suggest 50–80% of N_DEP_ is retained by the canopy (e.g. Sievering *et al*., 2007). ^15^N tracer recovery‐based methods tend to suggest lower CNU, from 50% maximum uptake across the canopy (MacKlon *et al*., 1996) to 25–30% (Friedland *et al*., 1991; Ammann *et al*., 1999) or as low as 2–5% (Lumme, 1994; Wilson & Tiley, 1998). These estimates are often difficult to interpret because of varying application methods, experimental systems, N_DEP_ magnitudes (Chiwa *et al*., 2004) and timescales, as well as species and site specific effects. They are also rarely compared against equivalent soil‐targeted deposition, making the separation of causative factors difficult [(see Lumme, 1994) for the only exception we could identify, albeit in an artificial microcosm]. Similarly, only a single study (Dail *et al*., 2009) reports a field‐scale ^15^N canopy fertilization. In their study, ^15^N recovery was high in plants (31% of ^15^N‐NH_4_, 61% of ^15^N‐NO_3_), but the implied ΔC/ΔN was modest due to high recovery in low C/N compartments such as bark (apparent retention in this pool is also complicated by the potential for abiotic retention on bark surfaces and incorporation into bark surface mosses and lichens). The C/N stoichiometry of this pool also varies between species (Allison, 1965), and hence, C effects from retention in bark may vary. Finally, both ^15^N isotope trace and unlabelled N addition experiments frequently apply a total N concentration far in excess of ambient N_DEP_ concentrations. This means it is difficult to interpret the effects of such experiments due to both positive and negative concentration‐dependent effects on ecosystem health as cumulative N inputs move towards N saturation (McNulty *et al*., 1996) as well as those specifically on canopy physiology (Maurice & Crang, 1986; Wellburn, 1990; Sievering *et al*., 2007; Wortman *et al*., 2012). Physiological effects on the canopy may occur earlier under foliar N loads due to CNU, especially if N inputs are in rare, high concentration events.

A second limitation to conventional ^15^N deposition treatments is that these typically apply a mineral ^15^N, typically NH_4_NO_3_, over the short term. In the real world, such inputs typically provide a minority of total N nutrition to plants in natural systems. Even under heavy N_DEP,_ internal litter mineralization remains the major source of N nutrition (Schulze, 2000; Högberg, 2012). Plant N uptake was historically thought to require complex organic precursors to be decomposed fully to ${\mathrm{NH}}_{4}^{+}$ and ${\mathrm{NO}}_{3}^{-}$, but there is now substantial evidence [see Näsholm *et al*. (2009) for a review] for organic N uptake by plants. Therefore, we were also interested if N from this source was partitioned in the same way as N from deposition.

We designed an experiment utilizing Sitka spruce [*Picea sitchensis*, (Bong.) Carr.] saplings, where a ^15^N signal could be traced from CNU, from conventional direct‐to‐soil mineral fertilization sources and also from labelled litter. We aimed to apply sustained N deposition inputs more regularly and for a longer duration than the few month durations and occasional inputs typical of other foliar ^15^N experiments. We applied very low total concentrations of N at high ^15^N enrichments in order to study the system at close to background N deposition levels. We utilized treatments with an artificial litter layer with high ^15^N enrichment and unlabelled N deposition to trace ^15^N from the litter under our deposition treatments.

We aimed to test the hypotheses that (i) the total return of ^15^N from deposition in aboveground parts of trees would be greater when ^15^N was applied to the canopy, rather than the soil; (ii) the proportional recovery of ^15^N in different tree organs (foliage, branches, stems and roots) and soil pools (SMB and bulk soil) would differ because of these two forms of application; and iii) ^15^N released from litter mineralization would have a greater recovery in plant pools compared to ^15^N applied in soil fertilizer treatments due to increased plant uptake of N from this source.

## Materials and methods

### Study site

Our study consisted of 3‐year‐old Sitka spruce saplings, located at Forest Research Northern Research Station, Scotland (55°86′N, 3°20′W). Thirty selected individuals from a cold‐stored (4 °C, lifted January 2011) batch of 2‐year‐old saplings were potted in 60 L pots on a mix of 90% homogenized stagnohumic gley topsoil (Clement, 2004) and 10% low N/P/K compost. The soil was collected from Griffin Forest, a Sitka spruce plantation in central Scotland (56°37′N, 3°47′W) and after mixing had a mean N content of 0.64% by dry weight. The C/N ratio of the soil mix was 24:1. In June 2011, the seedlings were randomly arranged in a 0.5 m spaced grid of 5 by six trees, surrounded in an overall 9 by 6 grid by an additional 60 trees which served to provide an edge around all treatment groups, and left to establish until summer 2012. Annual precipitation at the site was 704 mm, while mean monthly temperatures varied between 1 and 19°C. The trees remained in the initial configuration for the duration of the experiment.

### Experimental treatments

**Figure 1:**
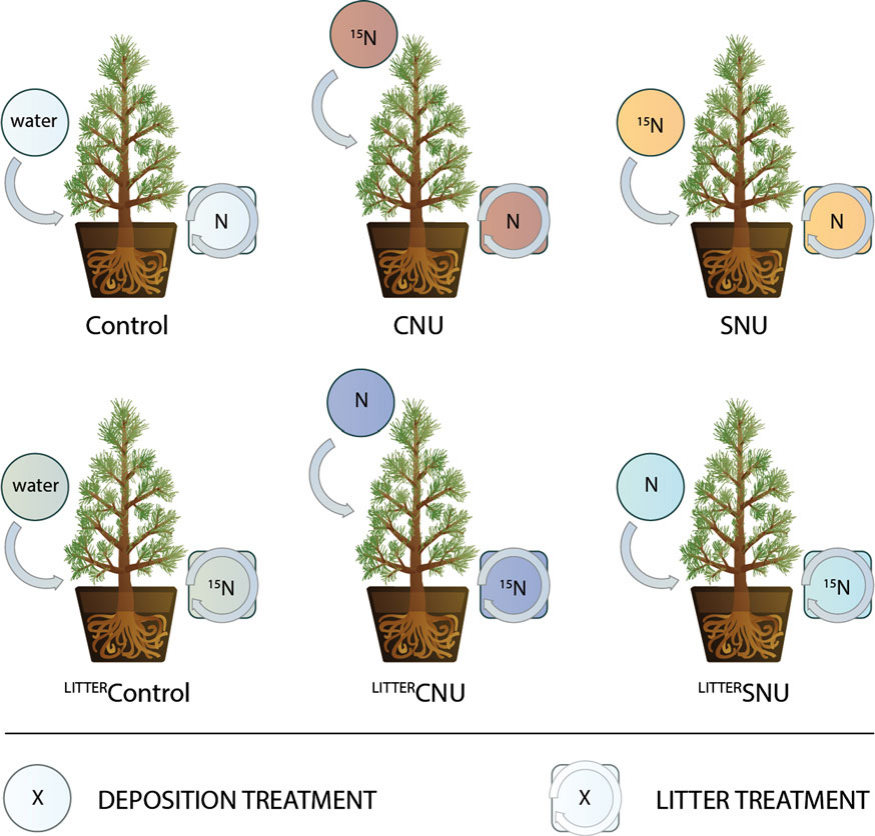
Treatment descriptions for the six experimental treatments. Each treatment received 0–1 sources of enriched ^15^N, either no enrichment (CONTROL), ^15^N‐enriched litter (^LITTER^CONTROL,^LITTER^SNU,^LITTER^CNU) or 98% double‐labelled ^15^N in deposition, that is ^15^ NH_4_^15^ NO_3_ (SNU, CNU). All deposition treatments received a total of 54 g N ha^−1^ yr^−1^ in deposition (as ^15^ NH_4_^15^ NO_3_ or NH_4_ NO_3_).

Individual trees were assigned to six treatments stratified based on current basal diameter and height as well as a series of soil CO_2_ efflux measurements made over spring and summer 2012 with an a EGM‐4 CO_2_ IRGA (PP Systems, Amesbury, MA, USA), as a proxy for differences in below‐ground nutrient cycling potential. Due to an aphid infestation in spring 2012, six trees had lost some of the 2011 cohort of needles and were each assigned to different treatments to avoid systematic biases. This stratification was designed to ensure an even mixture of tree size and apparent health in each treatment (*n* = 5 per treatment). In August 2012, we assigned the trees to their treatment groups and applied a layer of artificial litter to the soil. This artificial litter was obtained from the Gisburn Forest tree species trial, Lancashire, UK (54_01′30″N, 2_22′57″W) where 6–7 m tall *Sitka spruce* trees had been stem injected with ^13^C and ^15^N double‐labelled aspartic acid (*n* = 3, see Churchland *et al*., 2012). The labelled trees were felled in November 2010, and three natural abundance control trees were harvested from the same site in January 2011. All biomass was dried in a 70 C oven until needles were easily separated from the branches, then stored in paper sacks in a dry polytunnel until deployment. A random sample of 100 needles from each of the trees was measured for ^14^/^15^N and ^12^/^13^C isotope ratios 1 month before deployment, using the same methodology as later samples (see below). In the control trees, *δ* ^15^N was at natural abundance (0%). The labelled trees had mean foliar ^15^N atom % excess of 0.263, 0.850 and 1.231% over natural abundance, and a mean foliar N content of 1.12 ± 0.2 (SD) % and foliar C content of 48.88 ± 5.3 (SD) %. Total N and C concentrations were not significantly different (anova) among the source trees nor between injected and uninjected trees. Each potted mesocosm tree received 0.8 kg of litter (either ^15^N enriched or natural abundance), from a single randomly assigned source tree [to reduce the potential for interactive effects of litter mixing, for example Gartner & Cardon (2004); Smith & Bradford (2003)], in a single 4–5 cm deep layer. This amount of litter was selected to provide a reasonably deep and mixable litter layer across the pot surface. The six treatments (Table 1, Fig. 1) were designed to test a unique combination of ^15^N source and deposition type to the trees, varying in (a) litter type; either natural abundance or ^15^N‐enriched (indicated in treatment names by the superscript ^LITTER^); (b1) deposition type; either, N_DEP_ to the soil (SNU; soil nitrogen uptake) or N_DEP_ to the canopy (CNU; canopy nitrogen uptake); and (b2) level of ^15^N enrichment of the deposition treatment; either water control applied directly to the soil, (CONTROL), natural abundance 0.3663 atom % ^15^N (in the case of the ^LITTER^ treatments) or 98 atom % ^15^N (the source of ^15^N in all treatments without the ^LITTER^ prefix). All treatments had a single enriched ^15^N source (e.g. the ^LITTER^SNU treatment contained ^15^N‐labelled litter paired with an unlabelled N_DEP_ treatment to the soil), with the exception of the water control on unlabelled litter, which had no enriched ^15^N source.

**Table 1:** Treatment descriptions for the six experimental treatments. Total N_DEP_ for all deposition treatments was 54 g N ha^−1^ yr^−1^ applied as NH_4_NO_3,_ and ^15^N‐enriched treatments were 98 atom % ^15^N as ^15^NH_4_^15^NO_3_

Treatment ID	Litter	Type of application to soil	Type of application to canopy
CONTROL	Natural abundance	Water	–
^LITTER^C	^15^N‐enriched	Water	–
SNU	Nat. abun.	^15^N‐enriched N_DEP_	–
CNU	Nat. abun.	–	^15^N‐enriched N_DEP_
^LITTER^SNU	^15^N‐enriched	Nat. abun. N_DEP_	–
^LITTER^CNU	^15^N‐enriched	–	Nat. abun. N_DEP_

The simulated N deposition applied to 4 of the 6 treatments was equivalent to approximately 54 g N ha^−1^ yr^−1^ in excess of background N_DEP_ of either 98 atom % ^15^N as ^15^NH_4_^15^NO_3_, or unlabelled NH_4_NO_3_. We aimed to keep bulk N_DEP_ almost unaltered from ambient deposition and at the same magnitude in all N amendment treatments to allow a direct comparison between the foliar‐applied ^15^N label and the soil wet ^15^N deposition treatment with minimal effect of total N abundance relative to controls. Ammonium nitrate was chosen for the simulated N_DEP_, as it contains both mineral ions typically found in N deposition. This treatment was applied in deionized (DI) water solution every 3–6 weeks from February 2013 until March 2014 as either a 0.5 L (SNU applications), or a solution of 10–15 mL (CNU application), the canopy solution being increased in volume (but not in N or ^15^N content) in summer 2013 to match increases in the canopy biomass of the trees (based on observed growth). On average, each month, each treatment tree receiving a labelled deposition treatment (CNU and SNU) received 81 *μ*g ^15^N and each treatment tree with ^15^N‐litter (^LITTER^CNU and ^LITTER^SNU) received 81 *μ*g N at natural abundance. Soil treatments were sprayed onto the litter surface using a pressurized hand sprayer, while foliar applications were applied directly onto needles and twigs using a brush presoaked with treatment solution. This began at the top of the tree and continued on each branch in turn down through the canopy until the solution was exhausted, and visible drips of the solution onto the soil/litter during application were not observed. The bottle was then washed out with 10 ml rainwater and poured directly down the stem of the tree. Control water treatments (CONTROL and ^LITTER^C) were applied directly to the litter surface in the same manner and volume as the soil applications.

### Routine biomass measurements and maintenance

Routine measurements of tree growth were made every 2 months during the growing season and every 3 months outside the growing season. At each occasion, tree height was measured with a plumbline marked in centimetres, and basal diameter was measured as the mean of two calliper measurements at right angles across the stem at the litter surface. At each of these instances, deciduous leaf litter not derived from the experimental trees was removed and weeds growing in the pot were uprooted, manually shredded, and left on the soil surface, so none of the ^15^N in their foliage was removed from the potted system. The pots were free draining, so any ^15^N exported in flow through the pot was not retained.

### Time series measurements

Twenty‐five needles per tree were collected from the entire canopy on 13 occasions between August 2012 and May 2014, 10 of these being after the deposition treatments began in February 2013. This number of needles was chosen as a representative sample to avoid detrimental effects of cumulative defoliation on the trees. After bud burst in May 2013, a harvest of the 2013 needle cohort was made alongside the general harvest, which specifically targeted the 2011–2012 cohorts of needles. In the first instance (May 2013), the 2013 cohort sample was a single, entire bud, but subsequent harvests were taken from the entire current cohort of biomass in the same manner as the general needle harvest. For the May 2014 measurement (when the trees were destructively sampled), this sample was taken from the entire harvested needle biomass and contained more (~100) needles. All samples were collected immediately before application of the regular N deposition treatments, to allow as much time as possible for movement of the assimilation products within the tree (in contrast to short‐term foliar ^15^N recovery (e.g. Wilson & Tiley, 1998), and to allow maximum system retention of the ^15^N remaining on leaf surfaces by either canopy uptake or washing into the soil by rain events. The harvested needles were immediately transported to the laboratory and either immediately processed (see below), or frozen at −4°C until it was convenient to dry them (usually within 7 days).

Branch and twig samples were collected twice (October 2013 and at the end of the experiment in March 2014), but only analysed in March 2014. In October, two random branches were removed per tree from (i) the current year cohort and (ii) the oldest age class of branches (which contained biomass from the 2011, 2012, 2013 and 2014 growing seasons). In March, three branches were selected from each of these two sections from the total harvest mass of the whole tree. Three – 0.75‐cm‐diameter discs containing the entire radial section (bark included) were cut from the entire length of each branch and used for isotope analysis, while the whole branch was dried to obtain dry weight. In March 2014, a similar method was used, but radial discs were collected from three different branches. Litter samples were collected every 3 months as a scoop from the litter surface and were a small fraction (< 5%) of the total litter in the pots.

### Destructive sampling

The experiment was terminated at the end of March 2014, prior to the commencement of the year's growing season. After recording basal diameter and height, the mainstem was cut at the base to kill the tree and the branches immediately removed with clippers at their junction with the stem, then separated into the two age classes (2013 and older cohorts). The stem was also separated into these two sections by cutting at the divide between annual growth stages. This resulted in sections which contained the vertical growth achieved during each year but did not separate the radial growth occurring across the whole stem length. All biomass was dried in paper sacks inside a 80 °C oven until mass loss had ceased (3 days). After drying, the needles were separated from the branches and each section was weighed. The litter layer on the surface of each pot was removed using a trowel and a 7‐cm‐diameter, 20‐cm‐deep (to the base of the pot) soil core was taken from each pot at a random location between the main stem and the edge of the pot. The soil cores were separated into root and soil components while moist, and the soil was homogenized. Roots were treated as a single pool irrespective of age class, unlike above‐ground biomass. Fifteen grams of homogenized soil was dried in an 80 °C oven to prepare the soil for total ^14^/^15^N measurement as well as water content calculations based on mass loss. A further 15 g dry weight, moist subsample was fumigated for 3 days with chloroform in a vacuum oven, then extracted in 45 mL 0.05 M K_2_SO_4_ for 3 h on a 220 rpm shaker along with an unfumigated control. The extract solution was freeze‐dried, and subsamples of the salt were analysed on a CN analyser for C and N content. The remaining salt was rehydrated, if necessary adjusted in volume to deliver an appropriate amount of N for mass spectrometry when dried, and concentrated via diffusion using the PTFE‐enclosed acidified paper discs method (Stark & Hart, 1996). These discs were analysed on a SerCon Ltd. isotope ratio mass spectrometer (University of Aberdeen) for ^14^/^15^N ratio and N concentration.

Soil microbial biomass (SMB) N was calculated using N content and a K_*EN*_ conversion factor of 0.54 (Brookes *et al*., 1985), where SMB N was (total N extracted from fumigated soil/total N extracted) * K_*EN*_. Microbial ^15^N was calculated from these measurements along with *δ* ^15^N of the control and fumigated pools by:

(1) $$\frac{\delta^{15}N_{SMB}\;=\;\delta^{15}N_{fumigated}*N_{fumigated}-\delta^{15}N_{unfumigated}*N_{unfumigated}}{N_{fumigated}-N_{unfumigated}}$$

### Sample processing

All biomass samples (needles, wood and roots) were washed in distilled water to remove remaining surface residues and dried in a 80 °C oven until mass loss had ceased (usually 1–2 days). Needles on the branches and twigs were removed after drying and before milling. The samples were milled on a Retch MM‐200 ball mill, in metal capsules with a single ball, until a fine powder was produced, except for the needle samples between August 2012 and February 2014, which, due to their small volume, were milled in plastic microtest tubes with two small ball bearings. A subsample of this powder (∼3 mg) was analysed for [N], ^14/15^N, [C] and ^12/13^C on the isotope ratio mass spectrometer, along with standards of known isotope abundance.

### Experimental calculations and statistical analyses

We used the dry masses of the whole tree sections from March 2014 to calculate growth metrics to compare trees and to calculate an aboveground mass balance at the termination of the experiment. Rather than compare raw mass of the tree compartments, we calculated four metrics, bulking the labelled and unlabelled N deposition type combinations together to produce three treatments for bulk N regime with *n* = 10 [water control (CONTROL), foliar deposition (CNU), soil deposition (SNU)] to test these growth effects. The metrics calculated were above‐ground biomass (AGB, the sum of both the 2011–2012 and 2013 needle, branch and stem cohorts), AGB % Canopy (the percentage of the total biomass made up by the canopy), Canopy % Foliage (the percentage of the canopy biomass that was foliage) and height increment (length of leader at the end of the 2013 growing season as a percentage of height of tree at the end of the 2012 growing season).

The isotope analyses were performed on all six treatments where the source of ^15^N enrichment differed, initially with five replicates (i.e. *n* = 5 each, but reducing to 4 for the two treatments (CONTROL and ^LITTER^SNU) where one plant was removed). Differences in *δ* ^15^N were analysed as linear mixed effect models where the fixed effects were time and treatment while individuals were random effects. We fitted an autoregressive moving average correlation structure to account for autocorrelation of individuals through time, using the REML method due to small sample sizes. We also allowed for a greater variation in ^15^N concentration later in the experiment (due to the greater cumulative application of N) by implementing a variance structure. Comparisons between treatments in these models were performed by the Tukey HSD post hoc test, and models were compared with the modified AIC (Akaike information criterion) for small sample sizes, AICc.

The mass balance was calculated using the March 2014 biomass [N], and ^15^N measurements, assuming that all enrichment above natural abundance was derived from the experimental treatments. Belowground compartments (litter, soil, roots, microbial extracts) were not included in the mass balance as they were not fully harvested at completion of the experiment leaving some uncertainty over their absolute masses. The mass balance calculation was made by subtracting the atom % ^15^N in CONTROL from observed atom % in the five ^15^N‐enriched treatments, to calculate ^15^N_*excess*_, using the total mass of ^15^N added in the deposition treatments, or estimated to be released from the litter based on a separate litterbag experiment (R.K.F. Nair *et al*., unpublished data), ^15^N_*added*_ and the average N mass of the pool in question (N). We used equation 2 to work out the total ^15^N recovery, ^15^N_*recovery*_ (%), with uncertainty propagated fully to take into account uncertainty in original measurements and averages.

(2) $${}^{15}N_{recovery}\;\left(\%\right)\;=\;\frac{{}^{15}N_{excess}*N}{{}^{15}N_{added}}*100$$

### Overall ΔC/ΔN calculations

To calculate ΔC/ΔN from our treatments, we modified the calculations from the (Nadelhoffer *et al*., 1999) meta‐analysis of tracer studies. These calculations estimate an overall C effect of N_dep_ by partitioning total N_DEP_ inputs between different ecosystem pools based on observed ^15^N tracer return. The N_DEP_ assigned to each pool was multiplied by the C/N ratio of the pool to generate an overall C effect due to the N_dep_ inputs, which can be divided by the total N_dep_ to estimate a ΔC/ΔN effect. We used the same C/N values as (Nadelhoffer *et al*., 1999), which are generous towards tree pools (tending to be high), and applied them to simple, generalized (woody, nonwoody and soil) biomass pools. We altered the N assignment among tree and soil pools to match the values we calculated in the mass balance for the canopy‐targeting (CNU) and soil‐targeting (SNU) ^15^N deposition treatments, defining woody biomass as all stem wood and 2011–2012 branches, and nonwoody biomass as all needles and 2013 branches. We split the remaining N not acquired by the tree in the same ratio (7:1) between soil, and leaching and gaseous losses as found in Nadelhoffer *et al*. (1999). In the case of the CNU treatment, this assumed that exposure of the canopy to excess ^15^N did not affect soil partitioning of the isotope (due to background ambient N_DEP_, all treatments received a canopy load of N) and that expressed isotope abundance in the tree in this treatment included contributions from both potential canopy uptake and via the roots from the soil.

## Results

### Needle time series

**Figure 2:**
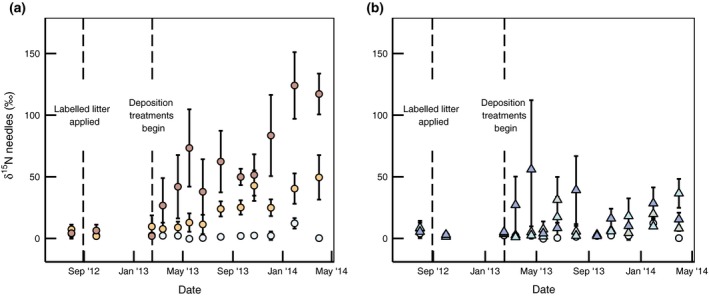
*δ* ^15^N (%) of needles older than the 2013 cohort from ^15^N‐labelled deposition treatments (a) and ^15^N‐labelled litter treatments (b). CONTROL is shown on both plots (white circles); on (a), plot treatments are CNU (red circles) and SNU(orange circles); and on (b), plot treatments are ^LITTER^CNU (dark blue triangles), ^LITTER^SNU (light blue triangles) and ^LITTER^C (grey triangles). Error bars show standard error of the mean (*n* = 5).

Differences in ^15^N concentrations between the ^15^N‐labelled N_DEP_ treatments (CNU and SNU) and the water control were apparent within 1 month of the deposition treatments beginning (Fig. 2). The *δ* ^15^N of needles in cohorts present before initiation of the experiment (2011–2012) in the ^15^N deposition treatments increased over time, to about 120% (CNU) or 38% (SNU) by April 2014, while the CONTROL treatment remained consistently close to natural abundance (~−3.5%). Over this period, the corresponding needles in the ^15^N litter treatments (^LITTER^C, ^LITTER^SNU, ^LITTER^CNU) did not display a trend in enrichment, although variability was very high in these treatments, especially early in the growing season. Treatment, date and the treatment:date interaction were all significant (*P* < 0.001) in explaining changes in ^15^N enrichment. CNU (post hoc Tukey HSD, *P* < 0.001) was significantly different than all other treatments, and SNU (*P* < 0.05) was significantly different from the CONTROL, but not the other ^15^N treatments (Table 2). In the most parsimonious model, the correlation structure did not improve the model fit.

**Table 2:** Tukey HSD comparisons among treatments in the most parsimonious mixed effect model for the 2011–2012 cohort needles ^15^N abundance over time

	CONTROL	^LITTER^C	^LITTER^SNU	^LITTER^CNU	SNU
^LITTER^C	0.06	/	/	/	/
^LITTER^SNU	0.16	−0.11	/	/	/
^LITTER^CNU	0.08	0.02	0.08	/	/
SNU	0.42 *	0.36	0.25	0.33	/
CNU	1.08***	1.02***	0.91***	0.99***	0.66***

The numbers in the table give the mean difference between each set of treatments (columns–rows).

Significance at *P* < 0.05 level indicated by *, at *P* < 0.001 level by***.

**Figure 3:**
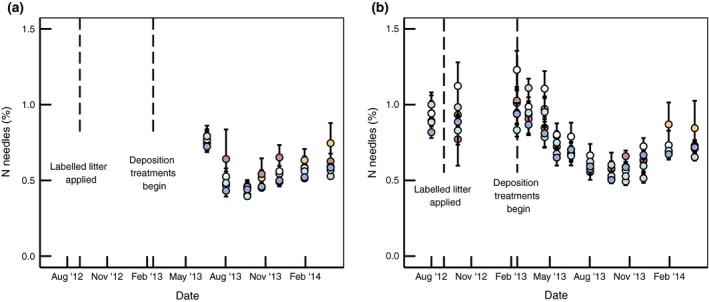
N content by dry mass (%) of needles from all treatments from 2013 cohort (a) and 2011–2012 cohort (b). While a yearly cycle is observed, this does not differ between treatments. Treatments are shown with same symbology as Fig. 2. Error bars show standard error of the mean (*n* = 5).

N concentration of the 2011–2012 needles fluctuated with an overwinter peak in N concentration in both 2013 and 2014, although peak [N] was not as great in the second year. This periodicity was not observed in the 2013 cohort of needles, which had their highest N concentration (~0.75%) soon after budburst, but did not peak over the winter (Fig. 3).

**Figure 4:**
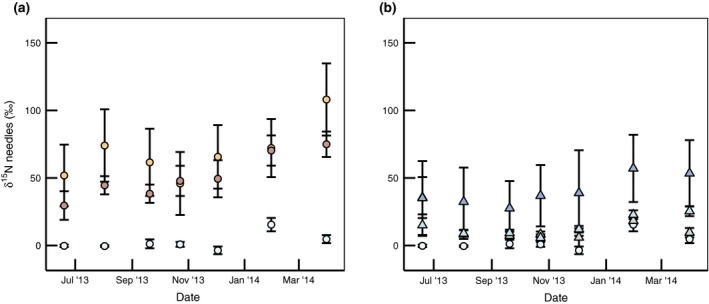
*δ* ^15^N (%) of 2013 needle cohort from ^15^N‐labelled deposition (a) and ^15^N‐labelled litter treatments (b). CONTROL is shown on both plots (white circles); on (a), treatments are CNU (red circles), and SNU (orange circles); and on (b), plot treatments are ^LITTER^CNU (dark blue triangles), ^LITTER^SNU (light blue triangles) and ^LITTER^C (grey triangles). Error bars show standard error of the mean (*n* = 5).

In the 2013 cohort of needles, the ^15^N enrichment was greater than the CONTROL in both CNU and SNU (Fig. 4a), and also in ^LITTER^CNU (Fig. 4b), although in this latter treatment this was due to a single individual which consistently displayed a high needle ^15^N enrichment. While this difference between treatments in the 2013 cohort of needles was significant (*P* < 0.05) along with time (*P* < 0.0001), there was no interaction term or correlation structure in the model with the lowest AICc, and the high variation in ^15^N concentration meant that only the SNU vs. CONTROL comparison was significant (post hoc Tukey HSD, *P* = 0.008) (Table 3).

**Table 3:** Tukey HSD comparisons among treatments in most parsimonious mixed effect ^15^N abundance model for 2013 cohort needle ^15^N abundance over time

	CONTROL	^LITTER^C	^LITTER^SNU	^LITTER^CNU	SNU
^LITTER^C	0.003	/	/	/	/
^LITTER^SNU	0.004	0.001	/	/	/
^LITTER^CNU	0.013	0.009	0.009	/	/
SNU	0.023**	0.02^a^	0.020^b^	0.011	/
CNU	0.017	0.014	0.019	0.014	−0.006

The numbers in the table give the mean difference between each set of treatments (columns–rows).

Significance at *P* < 0.01 level indicated by**.

Borderline significant differences are represented by *a*, indicating *P* = 0.086, and *b*,* P* = 0.072.

### Destructive harvest

The two trees which died were in the CONTROL, and ^LITTER^SNU, reducing their sample size to 4 from autumn 2013 until the destructive harvest. At harvest, there were no significant differences among the groupings of N treatments (CONTROL ^LITTER^CONTROL *n* = 9, SNU/^LITTER^SNU *n* = 9, CNU ^LITTER^CNU *n* = 10) in any of the four above‐ground biomass variables. Across the whole experiment, above‐ground biomass was 370 ± 119 g (SD) per tree, 74 ± 5 (SD) % of this being canopy, while the canopy was 38 ± 2 (SD) % needles by mass. The trees gained on average 12.4 ± 5.8 (SD) % of their initial height over the 2013 growing season, while litter mass at the end of the experiment was 65.5 ± 33.3 (SD) % of the original dry mass applied.

The ^15^N content of the major above‐ground biomass components (Table 4 and Table 5) varied among treatments. There were significant treatment effects in both 2013 (*P* < 0.005) and 2011–2012 (*P* < 0.001) needle cohorts, stem cohorts (*P* < 0.001, *P* < 0.001) and the 2013 branch cohort (*P* < 0.005), but not the 2011–2012 branch cohort. CNU significantly differed from the other treatments in the stem and branch sections (*P* < 0.001), as well as the 2011–2012 needles (*P* < 0.001). In both the 2011–2012 and the 2013 needle cohorts, all five ^15^N labelled treatments were significantly different from the CONTROL (*P* < 0.001) at harvest.

**Table 4:** Mean *δ* ^15^N % in 2011–2012 cohort tree compartments after 16 months

	Needles	Branches	Stem	Roots
CONTROL	0.2 ± 2^a^	0.5 ± 0^a^	27.5 ± 5^a^	4.6 ± 3
^LITTER^C	8.1 ± 2^b^	13.6 ± 13^a^	45.9 ± 11^a^	27.4 ± 4
^LITTER^SNU	36.5 ± 12^b^	4.5 ± 4^a^	268.2 ± 8 6^a^	77.7 ± 24
^LITTER^CNU	15.5 ± 5^b^	8.7 ± 5^a^	32.6 ± 7^a^	41.1 ± 46
SNU	36.4 ± 13^b^	0.4 ± 0^a^	111 ± 25^a^	67.5 ± 40
CNU	117.2 ± 17^b^	18.1 ± 13^b^	354.1 ± 77^b^	22.8 ± 10

Values shown ± standard deviation (*n* = 5).

Lowercase letters indicate significant differences (Tukey HSD) among treatments for the same pool at the *P* < 0.05 level or higher.

**Table 5:** Mean *δ* ^15^N % in 2013 cohort tree compartments after 16 months

	Needles	Branches	Stem
CONTROL	4.7 ± 3^a^	4.2 ± 3^a^	3.9 ± 1^a^
^LITTER^C	9.5 ± 3^b^	48.0 ± 35^a^	19.7 ± 21^a^
^LITTER^SNU	25.4 ± 4^b^	48.5 ± 26^a^	59.4 ± 44^a^
^LITTER^CNU	53.4 ± 25^b^	25.7 ± 16^a^	23.9 ± 10^a^
SNU	97.7 ± 32^b^	32.5 ± 32^a^	100.1 ± 64^a^
CNU	74.9 ± 9^b^	221 ± 37^b^	569.1 ± 167^b^

Values shown ± standard deviation (*n* = 5).

Lowercase letters indicate significant differences (Tukey HSD) among treatments for the same pool at the *P* < 0.05 level or higher.

Root *δ* ^15^N was not statistically different among treatments [mean N % was 0.55 ± 0.15 (SD), mean *δ* ^15^N 40 ± 36% (SD)], and although *P* was > 0.05, the lowest mean *δ* ^15^N was found in the CONTROL [4 ± 3% (SD)] and the highest *δ* ^15^N in the N manipulation (SNU, CNU, ^LITTER^SNU, ^LITTER^CNU) treatments (Table 4). Likewise, total soil *δ* ^15^N did not statistically differ among treatments [mean soil *δ* ^15^N was 38.1 ± 37% (SD)], but mean *δ* ^15^N % was lower in the CONTROL [*δ* ^15^N 16.9 ± 20 (SD)] than the labelled treatments [combined mean *δ* ^15^N 42.9 ± 39% (SD)]. Individually, CNU *δ* ^15^N [41.4 ± 48% (SD)] was lower than SNU *δ* ^15^N [59.2 ± 21% (SD)], but this difference was small and nonsignificant.

There were no statistical differences in total N extractable from 0.05 M K_2_SO_4_ unfumigated extracts between treatments (mean = 0.010 mg g^−1^ dry soil), nor between the N content of microbial biomass (*P* > 0.05), which was highly variable and estimated at 0.041 ± 0.03 mg g^−1^ soil. Microbial *δ* ^15^N calculated from the fumigations was very variable and was not related to treatment, and there was also no statistical difference between treatments in bulk soil ^15^N.

### Mass balance estimates of aboveground ^15^N recovery

The highest above‐ground recoveries of the ^15^N label from the mass balance were 60.14 ± 5.7% (SD) in CNU and 20.28 ± 6.9% in SNU. These two treatments recovered quantities of ^15^N significantly (*P* < 0.001 CNU, *P* < 0.01 SNU, respectively) greater than CONTROL (Table 6). ^15^N recovery was highest in the 2013 and 2011–2012 needles, and 2011–2012 stems in these treatments.

**Table 6:** ^15^N recovery as % of total applied ^15^N in the aboveground sections (*n* = 5 for each section) of the two labelled deposition treatments

	CNU	SNU
2013 Needles	7.17 ± 3.04%	9.50 ± 3.40%
2011–2012 Needles	13.41 ± 2.40%	3.94 ± 2.66%
2013 Branches	20.77 ± 2.86%	4.82 ± 3.46%
2011–2012 Branches	3.12 ± 0.14%	0.02 ± 0.07%
2013 Stem	1.04 ± 0.04%	0.21 ± 0.02%
2011–2012 Stem	14.64 ± 3.15%	2.78 ± 4.04%
Total Woody Biomass	18.80 ± 3.15%	3.01 ± 4.04%
Total Above Ground	60.14 ± 5.75%	21.28 ± 6.85%

Also presented are total accountancy in woody sections (stem and 2011–2012 branches, but not 2013 branches) and total ^15^N recovery aboveground. Errors terms are standard deviation obtained by propagating the error in measurements of different pools while total recovery and error are obtained by summing the recovery and propagating the error of individual pools making up the total.

When we calculated litter N release based on field litterbag decomposition rates (R.K.F. Nair *et al*. unpublished data) and total mass of litter applied, calculated ^15^N recovery was very low and never significantly different from the CONTROL. Even when we revised these low rates based on more conservative literature values (Titus & Malcolm, 1999; van Huysen *et al*., 2013), these remained small, displaying a low gross aboveground ^15^N return and high uncertainty (^LITTER^C 9.62 ± 6.54%, ^LITTER^SNU 12.59 ± 6.4% and ^LITTER^CNU 11.52 ± 6.1%) and were not taken forward to calculate ΔC/ΔN, nor shown in Table 6.

### ΔC/ΔN calculations

When propagated, the overall C sink effect from SNU was 44.2 ± 18 kg C kg N^−1^, split between trees and soil (Table 7). This sink was similar to that from Nadelhoffer *et al*. (1999) and had a large standard deviation (18 kg C kg N^−1^), which was mainly due to the high uncertainty on stem assignment in this treatment (Table 6). In contrast, the ΔC/ΔN estimate from CNU was 113.9 ± 16 kg C kg N^−1^, more than double the estimates from Nadelhoffer *et al*. (1999), and more than 2.5 times the one drawn from our soil treatment. This was due to a high overall N return (46% of N in nonwoody pools and 18% in woody pools, Table 6), and an assumption of correspondingly lowered ^15^N assignment to the soil, resulting in a slightly smaller soil ΔC/ΔN.

**Table 7:** Values of calculated ΔC/ΔN effect following (Nadelhoffer *et al*., 1999) and from the results of the two labelled deposition treatments of this experiment

	Meta‐analysis of soil ^15^N applications (Nadelhoffer *et al*., 1999)	Soil deposition (SNU)	Canopy deposition (CNU)
Tree	28.8	23.8 ± 18	104.6 ± 16
Soil	21	20.4 ± 2	9.4 ± 2
Total	49.8	44.2 ± 18	113.9 ± 16

The overall budgets presented in Nadelhoffer *et al*. (1999) were adjusted by altering the woody and nonwoody pools to match the values measured in our experiment (Table 6), with additional N drawn proportionally from soil (forest floor + mineral) and leaching + gaseous losses. Our woody pools were both stem pools, and 2011–2012 branches, while our nonwoody pools were the needle pools and 2013 branches. Errors are standard deviations from our study propagated with C/N ratios of Nadelhoffer *et al*. (1999).

## Discussion

In this controlled mesocosm study, we find canopy‐targeted N fertilization (and hence ambient rates of CNU) can result in around three times as much ^15^N tracer recovery aboveground as soil‐targeted N fertilization. Within trees, we find a four times higher ^15^N retention in wood (Table 6), indicating that N from CNU may favour this high C/N biomass. Otherwise, ^15^N recovery in our soil‐targeted treatment is similar to meta‐analyses showing about 20% above‐ground ^15^N recovery, with < 5% in wood in field experiments (Nadelhoffer *et al*., 1999; Templer *et al*., 2012a) as represented in global models which build on or closely reproduce such isotope‐derived data (e.g. Thomas *et al*., 2013). Thus, our results show that by omitting canopy uptake of N at ambient deposition rates, common methodologies could substantially underestimate a woody response to N_DEP._

### Contribution of canopy nitrogen uptake to ΔC/ΔN

We are aware of only one study (Dail *et al*., 2009) using a ^15^N tracer in the field at the forest canopy scale. Results from most other experiments must be carefully interpreted as N_DEP_ interacts with multiple leaves and branches as it passes through the canopy (Boyce *et al*., 1996), and N partitioning between biomass pools with different C/N ratios may occur on alongside phenological cycles in nutrient content (Millard & Grelet, 2010). Many CNU experiments last less than a year (Bowden *et al*., 1989; Eilers *et al*., 1992; Wilson & Tiley, 1998), consider limited microcosm systems (e.g. Lumme, 1994) or single branches (e.g. Vose & Swank, 1990; MacKlon *et al*., 1996). Elsewhere ‘natural experiments’ with an ambient ^15^N source (Friedland *et al*., 1991; Ammann *et al*., 1999) may not recover ^15^N from the whole tree, preventing inference of total ^15^N recovery. Notably, Dail *et al*. (2009) found bark (including epiphytic mosses and lichen) to be a major sink (45% recovery of ^15^NO_3_), rather than higher C/N bolewood and calculated a low C effect (8 kg kg N^−1^). We could not reliably separate bolewood from bark due to the size of our trees, but high ^15^N recoveries were observed in the stem when most of the CNU treatment was applied to the canopy. A small amount of the isotope solution was washed down the stem by both the dilution of any residue left in the treatment bottles (to add the entire ^15^N dose to the system) as well as from the canopy by ambient rainfall, but we expect this to be only a small part of the total ^15^N added. Therefore, mobilization of N from CNU within the tree, rather than bark surface absorption (c.f. Reiners & Olson, 1984; Dail *et al*., 2009) likely contributed to this stem response.

Needle N dynamics and mobilization to the active internal N cycling pool are phenologically controlled in species such as Sitka spruce (Millard & Grelet, 2010). In conifers, N is stored overwinter in the previous year's needles (Millard & Proe, 1992), while deciduous species store N in stems and roots or inner bark (Millard & Grelet, 2010). Remobilization of this N may contribute 9–46 % of N for new shoot growth (Millard & Proe, 1992), independent of soil N supply (Millard & Proe, 1993; Weatherall *et al*., 2006b). As our needle N was relatively conserved over the 2013–2014 winter in both needle age cohorts (Fig. 3), the high endpoint stem recovery in CNU was unlikely to be due to seasonally mobilized ^15^N in the phloem rather than actual assignment to this pool. The high ΔC/ΔN from this treatment (113.9 kg C kg N^−1^) assumes transport within the tree and N attribution to wood growth where high C/N ratios drive the response.

For our ΔC/ΔN calculations, we clumped canopy interception and CNU together, assuming that all N_DEP_ was intercepted on canopy surfaces and could be acquired across the canopy. N not acquired by the canopy was assumed available for uptake via root pathways and allocated between soil and losses (leaching) as in SNU. This discounted uptake of ^15^N washed out of the canopy in the CNU treatment and subsequently taken up the roots as we could not distinguish these pathways via ^15^N isotope recovery. Total CNU would scale with canopy cover and N_DEP_ interception, which in our canopy treatment was effectively ~ 100%, and so, we may overestimate the ΔC/ΔN effect of wet deposition. Dezi *et al*. (2010) used 60% N retention by the canopy (Chopping *et al*., 2008) and 80% uptake of this N by CNU (Sievering *et al*., 2007) to incorporate CNU into the G'DAY model, while Gaige *et al*. (2007) calculated a canopy N retention of > 70%. The 114 kg C kg N^−1^ for CNU would drop to 78 kg C kg N^−1^ with 60% N retention by the canopy; approximately 60% increase over SNU. Additional sensitivity analyses of the 114 kg C kg N^−1^ ΔC/ΔN are robust to the assumptions made in modifying calculations from Nadelhoffer *et al*. (1999) by changing the gross ^15^N uptake observed and overall within‐tree partitioning, and are shown in supplementary materials S1.

### Soil system and litter ^15^N recovery

The canopy harvest at the close of the experiment indicated that some litter ^15^N had been taken up by trees as total ^15^N recoveries in both 2011–2012 and 2013 needles that were significantly greater than CONTROL in all three ^LITTER^ treatments. However, over time the ^LITTER^ ^15^N recovery above ground was low, with a large heterogeneity early in the experiment. This did not correlate with individual source trees nor treatment replicates, but ^LITTER 15^N may have been more heterogeneously distributed between biomass age classes than CNU or SNU and unable to be captured by the lower number of needle replicates at this time and lower cumulative ^15^N additions. Nonetheless, Weatherall *et al*. (2006a) found only < 2.5% of N released from litter was retained in Sitka Spruce seedlings and other experiments using labelled litter are sparse (Hatton *et al*., 2012), but find similar aboveground recoveries, for example 2% of *Fagus sylvatica* litter ^15^N (Zeller & Colin‐Belgrand, 2001) over 4 years. ^15^N‐labelled litters from evergreen species cannot be collected in a single seasonal litterfall event, and, in this study, were harvested from felled trees labelled in a previous experiment. This methodology produces litters that have not naturally senesced and hence may have higher C:N ratios than natural litterfall. Our litters were harvested in November and may have also been N enriched due to storage (Millard & Grelet, 2010). It is unknown how these differences in litter stoichiometry may have affected systematic ^15^N recovery.

Similarly, despite strong aboveground ^15^N response from CNU and SNU, our deposition ^15^N inputs were not statistically detectable in the soil or roots. Between SNU and CNU, this was particularly surprising as it was expected that more ^15^N would have been taken up by roots when applied directly to the soil. However, plant N is usually transported as amino acids and is present in the xylem because of remobilization from N stores such as needles (Millard, 1996), senescence or because of xylem–phloem–xylem recycling in the roots (Marschnert *et al*., 1997). CNU‐obtained N may therefore have been transported to the roots for recycling leading to similar ^15^N concentrations in the CNU and SNU treatments.

### How general are the results?

The low N_DEP_ magnitude employed here was chosen to match ^15^N release from litter and inorganic applications and allowed a ^15^N trace at concentrations close to ambient nitrogen deposition. Physiological effects of N_DEP_ are well known (Schaberg *et al*., 1997; Elvir *et al*., 2006), but understanding the C response is difficult at ambient N_DEP_ levels. N manipulations usually raise N inputs so a response can be detected, and even with doses close to ambient total inputs, application rates differ from ambient deposition (Lovett & Goodale, 2011; Thomas *et al*., 2013). Our treatment (54 g N ha^−1^ yr^−1^) was several orders of magnitude less than typical experimental N treatments (e.g. Wallenstein *et al*., 2006; Gaige *et al*., 2007; Metcalfe *et al*., 2013) and was designed to avoid physiological responses to the treatment. Templer *et al*. (2012a) found a negative correlation between N addition rate and ^15^N recovery, but the sum of our total (ambient + manipulation) N inputs is less than the smallest N amendment in this study making comparisons difficult. This may have led to our trees being N deficient; Binns *et al*. (1980) suggest that healthy spruce needles are > 1.2% N by dry mass, and needle N concentrations were lower in the second winter of our experiment (Fig. 3), although there were no signs of deficiency (such as needle decolouration) in most individuals. Hence, high recovery of ^15^N under CNU could be a result of low N availability and a relatively high ability to respond in biomass N content without limits imposed by other factors (Fleischer *et al*., 2013). Conifers may also only respond in photosynthetic capacity to N at deposition rates up to 8 kg ha^−1^ yr^−1^ (Fleischer *et al*., 2013)_,_ which if applicable to our trees, mean CNU could be replacing, rather than supplementing ambient N nutrition. We assumed that this response was not limiting in our calculations, and even if a limit on photosynthesis response is reached, ΔC/ΔN may still increase via other mechanisms. For example, below‐ground C allocation may decrease under N deposition (Nadelhoffer, 2000; Janssens *et al*., 2010) as N scavenging becomes more efficient. Testing differences in allocation would be possible using a CNU treatment where all throughfall is removed before reaching the soil system so only a canopy ^15^N source is available.

Both ^15^N studies (Templer *et al*., 2012a) and forest inventory data (Thomas *et al*., 2009; Templer *et al*., 2012b) also suggest that N effects on growth are species specific, and potentially limited in evergreen needleleaf trees (Thomas *et al*., 2009). This contrasts with the large inferred effect of CNU the *Picea sitchensis* saplings in our study. Proportion of woody biomass increases with tree age (Helmisaari *et al*., 2002; Peichl & Arain, 2007), but the annual growth increment is a progressively smaller proportion of total biomass (Stephenson *et al*., 2014). Thus, similar strong CNU ΔC/ΔN effects in mature trees depend on sustained allocation in the observed pattern as trees age.

Also, comparatively little is known about dry N_DEP,_ and it is also difficult to assess how CNU of wet and dry forms of N additions may compare if they differ in canopy interception and uptake capacity. Canopy uptake of these forms is likely to depend both on type of N input as well as individual species physiology; in the case of diffusion of ^15^ ${\mathrm{NH}}_{4}^{+}$ or ^15^ ${\mathrm{NO}}_{3}^{-}$ ions, this depends on leaf cuticle charge and/or stomatal conductance [see Sparks (2009)]. Differing rates of incorporation among tissues, such as between needle age classes in our experiment, could also be explained by differences in these factors. Future experiments with only labelled cations or anions, physiological stresses or alternate deposition methodologies could give further insight over these mechanisms.

### Implications for regional and global C sequestration

Aside from methodological concerns, we can still question how accurately uptake rates from experiments on single species can be scaled to compare with correlative analyses (e.g. Magnani *et al*., 2007; Thomas *et al*., 2009) and global models (e.g. Thomas *et al*., 2013). Most research on CNU has focused on wet deposition on evergreen conifers (e.g. Eilers *et al*., 1992; Lumme, 1994; Dail *et al*., 2009) and often on young trees (Bowden *et al*., 1989; Eilers *et al*., 1992; Lumme, 1994), and high correlative ΔC/ΔN responses come from widely distributed forests containing both broadleaf and evergreen trees, which may substantially differ in many relevant physiological traits (Adriaenssens *et al*., 2010).

Process‐based models provide a useful tool to upscale physiological understanding of mechanisms. Available global models representing the N effects on C uptake differ in their representation of plant N acquisition, with N uptake being driven by plant stoichiometry (Thornton *et al*., 2007), C assignment to roots (Zaehle & Friend, 2010) and N availability in soil pools. N uptake also occurs after soil immobilization demands are met (Gerber *et al*., 2010; Thomas *et al*., 2013). Such models are typically calibrated against long‐term N tracer and fertilization experiments where N is added to the soil (e.g. Thomas *et al*., 2013). These models may represent the fraction of N which reaches the soil surface, but if CNU is as substantial as indicated in our study, they may overestimate the size of N_DEP_ inputs to the soil, and simultaneously underestimate the total N acquired by trees. If this is the case the expected root uptake decreases (due to reduced soil N availability), but total tree N uptake increases via CNU (as this N is first available to trees). In addition, we showed that this N may be assigned to high C:N wood, enhancing the C sequestration effect from this alternative source of N over conventional patterns of allocation. Aside from variation due to species, deposition type and dosage effects, constraints on CNU may be stoichiometric or depend on physiological drivers (e.g. of ion exchange) and require additional work to fully understand.

Nonetheless, our study demonstrates experimentally that CNU may account for at least some of the increased C effect of N deposition shown in correlative studies (Magnani *et al*., 2007; Thomas *et al*., 2009; Ferretti *et al*., 2014) over conventional ^15^N experiments (Nadelhoffer *et al*., 1999; Templer *et al*., 2012a) and understanding N deposition purely in terms of its effect on the soil system may substantially underestimate ecosystem‐level N effects. We recommend that CNU be investigated at realistic levels and across representative species to understand its potential importance in forest C sequestration and the necessity to include this in large scale models. To properly assess the impact of N deposition on C sequestration, it is vital that real‐world conditions are represented in the experiments which inform this understanding.

## Supporting information


**Appendix S1** Materials.
