# Supplementary material for: Does canopy nitrogen uptake enhance carbon sequestration by trees?
Source: Glob Chang Biol. 2015 Dec 14;22(2):875–88. doi: 10.1111/gcb.13096 (PMC4738422; doi:10.1111/gcb.13096)
Supplement: Supplementary file 1 — Appendix S1 Materials. [file GCB-22-875-s001.docx]

SUPPLEMENTARY MATERIALS

SENSITIVITY ANALYSIS

The ΔC/ΔN calculation we perform in this paper modify the simple ΔC/ΔN calculations by Nadelhoffer *et al.* (1999) using two observations of the ^15^N recovery from our experiment:

1. a difference in gross ^15^N recovery between SNU and CNU
2. a difference in ^15^N partitioning between wood and non-wood N pools (with different C/N ratios) under SNU and CNU

These two components of the calculation assume (i) that the CNU treatment applications were realistic and the gross amount of the ^15^N label applied in the field was acquired across the canopy and assigned within the trees and (ii) that the high wood return from the CNU treatment was representative of mature stands and not an artefact of the young trees or methodology in this study. To test the robustness of our result, here we calculate a ΔC/ΔN using either one of these assumptions, otherwise using the calculations based on unmodified C/N ratios from a) Nadelhoffer *et al.* (1999) or b) our observed SNU treatment, which fairly closely matched Nadelhoffer *et al.* (1999). This results in various ways to recalculate ΔC/ΔN as shown in Table S1. Also shown are the % increases over the ΔC/ΔN calculated from SNU of each alternative scenario. The calculations are more sensitive to the total ^15^N return differences in ^15^N partitioning, but in all cases the calculated ΔC/ΔN are substantially greater than the 42 kg C kg N^-1^ calculated from the SNU treatment.

Table S1: Sensitivity analysis for ΔC/ΔN calculations from CNU treatment. Alternative scenarios are presented using modified gross ^15^N recovery in trees or internal ^15^N partitioning using alternative quantitites and ratios from the either Nadelhoffer *et al.* (1999) meta-analysis or SNU treatment.

| Gross Tree ^15^N Recovery | Within-Tree ^15^N Partitioning | ΔC/ΔN | Increase over SNU ΔC/ΔN (%) |
| --- | --- | --- | --- |
| CNU | CNU | 115 | 185 |
| Nadelhoffer (1999) | CNU | 56 | 38 |
| SNU | CNU | 56 | 38 |
| CNU | Nadelhoffer (1999) | 97 | 141 |
| CNU | SNU | 66 | 64 |
